# High-Sensitivity Terahertz Sensors for Sample Detection

**DOI:** 10.3390/s26102918

**Published:** 2026-05-07

**Authors:** Dongshan Wei

**Affiliations:** 1 College of Physics and Electronic Engineering, Hainan Normal University, Haikou 571158, China; dswei@hainnu.edu.cn; 2 Shenzhen Institutes of Advanced Technology, Chinese Academy of Sciences, Shenzhen 518055, China

## 1. Introduction

Terahertz (THz) sensors refer to a class of photoelectric detection devices that utilize electromagnetic waves in the frequency range of 0.1–10 THz to achieve non-destructive, label-free, and high-sensitivity measurement of physical, chemical, and biological parameters by analyzing the spectral amplitude, phase, resonance shift, or transmission variation. The core functions of THz sensors encompass high-sensitivity refractive index sensing [1,2], trace component detection [3,4], moisture and thickness measurement [5,6], non-invasive structural imaging [7], and multifunctional switching detection [8]. THz sensors can quantitatively characterize subtle changes in the permittivity and absorption coefficient, identify micro-traces of biological or chemical substances, monitor highly water-absorbent liquid samples through solvent engineering or microfluidic integration, and achieve self-calibration, dynamic tunability, and mode-selective sensing via structural design [9,10]. In contrast to near-infrared sensors that only detect surface information, they may offer a penetration depth of approximately several hundred micrometers, enabling true non-destructive characterization of internal compositions.

Currently, fundamental research on THz detection mechanisms, metamaterial design, and low-dimensional material-based sensing technologies has achieved remarkable progress in laboratories. Preliminary applications have been explored in public security inspection, industrial non-destructive testing, food safety monitoring, chemical analysis, and biomedical diagnosis. With the assistance of metamaterials [11,12], two-dimensional materials [13,14,15], micro–nano structures [16], and even artificial intelligence (AI) techniques [17], the detection sensitivity for liquid, solid, and gas samples has been effectively improved. However, several challenges restrict their practical application, such as the severe water absorption of THz waves, limited signal strength, high device costs, and insufficient anti-interference capability. Additionally, the weak spectral response of trace samples and incomplete fingerprint databases also hinder the popularization and standardization of THz sensing in actual sample detection scenarios [9].

The future development of THz sample detection will focus on high-sensitivity sensing, miniaturized integration, and intelligent identification. Advanced functional materials, such as two-dimensional materials and metasurfaces, will be continuously optimized to enhance THz wave–matter interactions and enable trace sample detection. Compact on-chip THz devices and portable detection modules will gradually replace traditional large-scale equipment [18]. Furthermore, the combination of machine learning and spectral big data analysis will facilitate fast, automatic, and high-precision qualitative and quantitative analysis of complex samples.

These advances collectively push THz sensing toward higher sensitivity, lower detection limits, stronger stability, and higher integration levels, demonstrating enormous application potential in chemical analysis, biomedical diagnosis, food quality control, environmental monitoring, and industrial on-line detection.

## 2. Overview of Published Papers

All submissions were evaluated based on their technical merit and relevance, and seven high-quality papers were ultimately accepted for inclusion in this Special Issue.

Below, we present the list of accepted contributions, each followed by a brief summary.

In contribution 1, the authors proposed a novel integrated quasi-optical THz liquid sensor by leveraging mode-parity-dependent interaction with a capillary-confined analyte. This sensor was fabricated via deep reactive ion etching, integrating couplers, quasi-optical lenses, Bragg reflectors, and a microfluidic capillary channel in one chip. Experimental validations with water and isopropanol demonstrated linear resonance frequency shifts and transmission loss changes corresponding to refractive index and absorption variations, with real-time compensation for system fluctuations. The sensor features compact on-chip integration, self-calibration capability, and compatibility with continuous in-line monitoring, outperforming conventional free-space THz sensing setups in stability and practicality. This proposed platform provides a robust solution for high-precision liquid characterization in chemical analysis, biomedical diagnostics, and industrial process monitoring.

In contribution 2, the authors demonstrated a non-destructive coffee bean characterization and sorting method using THz spectroscopy to overcome the traditional surface-only limitation of near-infrared techniques. Both transmission and reflection THz measurements were performed on green, roasted, and quaker coffee beans, revealing that their THz optical properties are dominated by their internal moisture content with a penetration depth of approximately 150 μm at 0.5 THz. Meanwhile, they developed a stray radiation correction algorithm to suppress diffraction artifacts from irregular bean shapes, and used the THz tomography to visualize internal structural inhomogeneity. Furthermore, they built a robot-guided THz scanning system to identify green and roasted beans in bulk samples. Their work enables the internal compositional evaluation of whole beans, offering an industrially feasible tool for moisture-based coffee sorting and quality control.

In contribution 3, the authors developed and systematically optimized a chirp-tunable THz source based on pulse front tilt in a MgO-doped LiNbO_3_ prism. They studied the influence of pump beam spot size on THz generation efficiency, spectral bandwidth, and chirp characteristics experimentally and theoretically. The results indicated that a larger pump spot diameter (3.7 mm) significantly enhanced THz output power—nearly three times that of a 1.8 mm spot—and broadened the spectral bandwidth by promoting frequency component superposition, thus weakening the chirp effect, while a smaller spot intensified the spatial chirp and narrowed the spectrum. This work provides a straightforward approach to tailoring THz wave chirp and power, supporting improved frequency, bandwidth, and intensity responses in high-performance THz sensors.

In contribution 4, the authors designed a reconfigurable dual-functional THz metasurface sensor using a Dirac semimetal and vanadium dioxide (VO_2_) for switchable sensing and filtering operations. By thermally controlling the insulator-to-metal phase transition of VO_2_, the sensor switched between a high-sensitivity refractive index sensor and a band-pass filter. In the insulating state, it operated as a sensor with a sensitivity of 374.40 GHz/RIU and a high Q-factor of 69.97 with polarization insensitivity. In the metallic state, it functioned as a band-pass filter centered at 2.01 THz with a 3 dB fractional bandwidth of 0.91 THz. The proposed integrated dual-function device enriches the design paradigm of THz systems, enabling compact and multifunctional platforms for communication and sensing applications.

In contribution 5, the authors proposed a label-free THz metasurface sensor for specific and sensitive detection of L-valine and L-phenylalanine aqueous solutions. They designed an asymmetrically folded dual-aperture metallic ring metasurface, adopted a water–glycerol mixture (volume ratio 2:8) as the solvent to reduce strong THz absorption by pure water, fabricated a customized sample chamber with a fixed liquid thickness of 15 μm to ensure stable measurement conditions, and constructed a vertically incident THz-TDS system for horizontal sample placement. The sensor achieved a concentration sensitivity of 9.98 GHz/(mmol·L^−1^) for L-valine, significantly higher than 1.88 GHz/(mmol·L^−1^) for L-phenylalanine, enabling specific recognition. This work solves the strong absorption problem of aqueous solutions in THz sensing, providing a reliable, label-free tool for trace amino acid detection.

In contribution 6, an ultra-high-sensitivity THz metamaterial sensor based on QBIC was proposed for low-concentration biological detection. A double-chain separated resonant cavity structure was designed, and symmetry breaking was introduced to excite the high-Q QBIC mode. The simulation showed a refractive index sensitivity up to 544 GHz/RIU after optimizing structural asymmetry to balance ohmic loss and quality factor. Experiments achieved ultra-low limits of detection: 0.0025 mg/mL (~12 μM) for lithium citrate and 0.03125 mg/mL (~0.47 μM) for bovine serum albumin, requiring only 20 μL of sample per test. The strong field confinement of the QBIC mode drastically enhanced light–matter interactions and the signal-to-noise ratio. This work establishes a high-efficiency platform for trace and micro-volume biological sample sensing, outperforming most reported THz metamaterial sensors.

In contribution 7, a dynamically tunable four-frequency refractive index sensor based on graphene metamaterial was proposed in the mid-infrared region. The three-layer structure (gold bottom, dielectric middle, patterned graphene top) supported four strong absorption peaks, three of which exceeded a rate of 98%, meaning perfect absorption. By electrically adjusting the Fermi energy of graphene, the resonance positions were flexibly tuned, and the relaxation time was found to effectively modulate absorption intensity. Owing to structural symmetry, the device exhibited wide-angle polarization insensitivity, especially for TE waves. The maximum sensitivity reached 21.60 THz/RIU with excellent linearity between resonance shift and refractive index change. This design achieves multi-frequency, tunable, and polarization-insensitive high-sensitivity sensing, offering a promising candidate for compact and adaptive optical detection systems.

## 3. Conclusions

In this Special Issue, we present seven outstanding contributions that advance high-sensitivity THz sensors for low-concentration substance detection. The aim is to highlight the state-of-the-art designs, working mechanisms, and practical applications in this rapidly developing and technologically critical field. This collection brings together global advancements in THz photonics, integrated devices, metamaterials, and on-chip sensing systems, showcasing highly original, innovative, and impactful breakthroughs in sensitivity, stability, and functionality. Collectively, these studies address key challenges including strong water absorption, limited signal-to-noise ratio, lack of self-calibration, and insufficient detection limits for trace and low-concentration samples. This Special Issue offers a comprehensive reference for understanding recent progress, defining current challenges, and guiding future directions in high-performance THz sensing technologies. I would like to express my sincere gratitude to all of the authors for their excellent contributions, as well as to the reviewers for their dedicated effort and professional insights, which were indispensable in ensuring the high quality and impact of this Special Issue.

## References

[1] Sheheryar T., Tian Y., Lv B., Gao L. (2025). High-sensitivity refractive index based terahertz metasurface biosensor for detecting multiple cancers and infectious diseases. Photonics Nanostructures Fundam. Appl..

[2] Abdollahvand M., Azadi A.H., Ebrahimifard A., Heidarzadeh H. (2025). A tunable graphene terahertz sensor with high sensitivity and figure of merit for refractive index biosensing. Sci. Rep..

[3] Wang R., Zhang D., Chen L., Zhang N., Li D., Jiang R., Zhang X., Yang X., Zhang L., Wang S. (2025). Ultra-compact broadband terahertz spectroscopy sensor enabled by resonant-gradient metasurface. Nat. Commun..

[4] Liu Q., Ning T.G., Li J., Zheng J.J., Liang L.J., Yan X., Yao H.Y., Wang Z.Q., Li Z.H., Hu X.F. (2025). Terahertz Metasurface Multidimensional Biosensor Based on Quasi-Bound States in the Continuum for Trace Analyte Detection. IEEE Sens. J..

[5] Hidayat R., Aftab S., Ali Z., Koyyada G., Khalid A., Hegazy H.H., Yahia I.S., Truong N.T.N. (2025). Materials and strategies for Terahertz humidity sensing. Microchem. J..

[6] Su K., Shen Y.C., Zeitler J.A. (2014). Terahertz Sensor for Non-Contact Thickness and Quality Measurement of Automobile Paints of Varying Complexity. IEEE Trans. Terahertz Sci. Technol..

[7] Nsengiyumva W., Zhong S., Zheng L., Liang W., Wang B., Huang Y., Chen X., Shen Y. (2023). Sensing and Nondestructive Testing Applications of Terahertz Spectroscopy and Imaging Systems: State-of-the-Art and State-of-the-Practice. IEEE Trans. Instrum. Meas..

[8] Zeng X., Peng K.-X., Li Y., Zhang H.-L., Kong B. (2025). Switchable and Multifunctional Terahertz Metasurfaces Based on Multiple Tunable Materials. Plasmonics.

[9] Ajayan J., Sreejith S., Manikandan M., Lai W.-C., Saha S. (2024). Terahertz sensors for next generation biomedical and other industrial electronics applications: A critical review. Sens. Actuators A Phys..

[10] Wang B., Wang H., Bao Y., Ahmad W., Geng W., Ying Y., Xu W. (2025). Sustainable Materials Enabled Terahertz Functional Devices. Nano-Micro Lett..

[11] Wang W., Sun K., Xue Y., Lin J., Fang J., Shi S., Zhang S., Shi Y. (2024). A review of terahertz metamaterial sensors and their applications. Opt. Commun..

[12] Li X., Sun J., You Q., Li Y., Deng T. (2025). Recent Progress in Novel Terahertz Metamaterial Sensors: A Review. IEEE Sens. J..

[13] Bi H., Yang M., You R. (2023). Advances in terahertz metasurface graphene for biosensing and application. Discov. Nano.

[14] Feng Y., Liang M., Zhao X., You R. (2025). Fabrication and modulation of flexible electromagnetic metamaterials. Microsyst. Nanoeng..

[15] Niu Y., Ding N., Wu W., Fu M., Hu J., Yu M., Guo Z., Zhou F., Ren X., Wang Y. (2025). Progress and prospect of terahertz detectors based on two-dimensional materials. Mater. Today Nano.

[16] Inomata N., Takahashi T., Sakai Y., Okatani T., Kanamori Y. (2025). Fabrication and application of 3D Terahertz metamaterials with vertical multinanogaps for spectroscopic sensing. Sci. Rep..

[17] Yu J., Pu H., Sun D.-W. (2025). An advanced deep learning-driven Terahertz metamaterial sensor for distinguishing different red wines. Chem. Eng. J..

[18] Ma L., Fan F., Shi W., Ji Y., Wang X., Chang S. (2024). Terahertz on-chip sensor based on Mach-Zehnder waveguide interferometer for selective recognition of reducing drug. Sens. Actuators A Phys..

